# Association of assisted reproductive technology and multiple pregnancies with the risks of birth defects and stillbirth: A retrospective cohort study

**DOI:** 10.1038/s41598-018-26567-2

**Published:** 2018-05-29

**Authors:** Min Yang, Xiao-Bo Fan, Jiang-Nan Wu, Ji-Mei Wang

**Affiliations:** 1 Department of Neonatology, Obstetrics and Gynecology Hospital of Fudan University, Shanghai, China; 2 Department of Clinical Epidemiology, Obstetrics and Gynecology Hospital of Fudan University, Shanghai, China

## Abstract

Assisted reproductive technology (ART) has been widely used among women with infertility. However, the association of ART with birth defects and stillbirth remains controversial and has rarely been reported in China. A retrospective cohort study of 112,043 pregnant women and 114,522 newborns from 2006 to 2016 was performed. Compared to spontaneously conceived infants, ART-conceived infants had a higher likelihood of any birth defect, with an adjusted odds ratio (OR) of 2.10 (95% confidence interval, 1.63–2.69). ART-conceived infants also had a significantly increased risk for subcategories of cardiovascular, musculoskeletal, urogenital, gastrointestinal, and respiratory defects. Most (62.25%) of the effect of ART on birth defects was a direct effect, whereas 37.75% of the effect of ART on birth defects was due to multiple pregnancies (i.e., an indirect effect). Compared with naturally conceived singletons, the combined effect of ART and twins on the risk of birth defects was lower than that of the sum of the individual effects of ART and twins on the risk of birth defects, with an adjusted OR of 0.54 (0.32–0.92). These findings clearly show that ART is associated with an increased risk of birth defects in China and may provide guidance to couples and obstetricians in selecting numbers of pregnancies and in identifying organs at a high risk of birth defects.

## Introduction

Assisted reproductive technology (ART), including *in vitro* fertilization (IVF) and intracytoplasmic sperm injection (ICSI), has been widely used among women with infertility^1–4^. However, whether ART is associated with an increased risk of birth defects remains controversial^5–8^. Even though several cohort studies and multiple systematic reviews or meta-analyses have found that ART treatment may be associated with an increased risk of birth defects^4,9–12^, some other studies have concluded that the observed increase in the risk of birth defects after ART may be attributed to infertility-related maternal characteristics^13–16^.

Since the first infant conceived by ART in China was born in 1988, many ART-conceived infants have been born^17^. To strengthen the management of ART treatment, the Ministry of Health of the People’s Republic of China released ART regulations in 2001 stipulating that the total number of eggs, zygotes and embryos per transplant cycle is limited to ≤3 for women aged 35 years and ≤2 for women aged <35 years^18^. Some studies have investigated the association between ART and birth defects in Chinese populations. In a national hospital-based retrospective study, ART-conceived infants were estimated to account for approximately 1.01% of total deliveries in China in 2011, and the author further found that ART was associated with an increased risk of maternal and neonatal complications (e.g., premature delivery, placenta previa, gestational diabetes mellitus, low birth weight, and infant mortality)^15^. Another multi-centre study of 15,405 ART-conceived infants found that 1.23% of the subjects had one or more major birth defects^6^. Several studies have compared the risk of birth defects between children conceived by IVF and children conceived by ICSI^6,19–22^. Liu *et al*. studied the risk of birth defects among ART-conceived children compared with that among spontaneously conceived children in a small sample size (8,240 naturally-conceived singletons and 567 ART-conceived births) and concluded that no statistically significant association existed^23^. However, little is known about the association of ART with the risk of birth defects based on a large sample size in China.

Similarly, the relationship between ART and the risk of stillbirth also remains controversial. A large Nordic cohort study did not find a significant association between ART and stillbirth^24^, but other studies have indicated that ART is associated with an increased risk of stillbirth^25,26^. However, few studies on the association of ART with stillbirth have been conducted in China.

Furthermore, multiple gestations seem to be more common among pregnancies conceived by ART compared to those conceived naturally^1–4^. In the United States, 1.7% of infants were conceived with ART, but more than 19% of twins and 25% of triplets or higher multiples were conceived with ART^4^. Multiple gestations are considered to be involved in the causal pathway between ART and birth defects^27^ and have been used to explain why the associations between ART and birth defects appear to be weaker among infants from multiple births than among those from singleton births^11^. However, few studies have investigated the relationship between multiple gestations and birth defects among infants from natural and ART-assisted conceptions.

China has abolished the “one child” policy and has allowed two children to be born in each family since 2015, which resulted in a substantial increase in the number of infants born to 18.46 million people in 2016, reflecting an 11.5% increase in births compared to that in 2015^28^. The change in the birth policy may have increased the demand for ART. Therefore, determining the associations of ART with birth defects and stillbirth and identifying the extent to which the association between ART and the risk of birth defects may be mediated by multiple gestations are important. We hypothesized that ART may be associated with slightly increased risks of birth defects and stillbirth and that the impact of ART on birth defects may be greater for infants from singleton births versus infants from multiple births. Therefore, we conducted a retrospective cohort study to evaluate these hypotheses.

## Results

### ART and birth defects

A total of 112,043 pregnant women and 114,522 newborns were included in the analysis. Among the pregnant women, 2.22% (2,484) had received ART treatment. Pregnant women who received ART treatment were older and were less likely to be nulliparous. A lower proportion of these women had singleton births compared to women who conceived spontaneously. Moreover, the women in the ART group were likely to have an abnormal pregnancy history and were at a higher risk for poor pregnancy conditions and developing pregnancy complications compared to women in the spontaneous conception group (Table 1).

**Table 1:** Characteristics of pregnancies and birth outcomes according to the mode of conception.

Characteristics of pregnancy	Spontaneous conception (N = 109,559)	Assisted conception (N = 2,484)	P value
	no. of pregnancies (%)		
Maternal age at delivery (years)*			<0.001
≤24	10,250 (9.4)	25 (1.0)	
25–34	89,682 (81.9)	1,629 (65.6)	
≥35	9,606 (8.8)	830 (33.4)	
Residence*			<0.001
Shanghai	90029 (82.2)	1928 (77.6)	
Other provinces	19476 (17.8)	556 (22.4)	
Gravidity (mean (SD))^¶^	1.7 (1.1)	1.8 (1.1)	<0.001
Nulliparous	92,007 (84.0)	1,597 (64.3)	<0.001
History of abnormal pregnancy	147 (0.1)	56 (2.3)	<0.001
Multiplicity			
Singleton	107,978 (98.6)	1,644 (66.2)	<0.001
Twin	1,541 (1.4)	822 (33.1)	
Triplets	40 (0.1)	18 (0.7)	
Maternal disease in pregnancy			
Gestational diabetes	3,753 (3.4)	270 (10.9)	<0.001
Pre-existing diabetes	3,216 (2.9)	186 (7.5)	<0.001
Anaemia	4,295 (3.9)	169 (6.8)	<0.001
Hypertension	654 (0.6)	50 (2.0)	<0.001
Pregnancy-induced hypertension	1,015 (0.9)	61 (2.5)	<0.001
**Birth outcomes**	**Births after spontaneous conception (N = 111**,**180)**	**Births after assisted conception (N = 3**,**342)**	**P value**
	**no**. **of births (%)**		
Gender^#^			
Male	57,533 (51.8)	1,705 (51.1)	0.426
Birth weight (g, mean (SD))^¶^	3306 (655)	2879 (984)	<0.001
Stillbirth	308 (0.3)	34 (1.0)	<0.001
Birth defect			
Any defect	1,031 (0.9)	77 (2.3)	<0.001
Cardiovascular defect	192 (0.2)	21 (0.6)	<0.001
Musculoskeletal defect	390 (0.4)	20 (0.6)	0.018
Urogenital defect	157 (0.1)	17 (0.5)	<0.001
Gastrointestinal defect	46 (0.0)	5 (0.1)	0.016^§^
Central nervous system defect	9 (0.0)	2 (0.1)	0.039^§^
Respiratory defect	4 (0.0)	2 (0.1)	0.012^§^
Chromosomal defect	54 (0.0)	3 (0.1)	0.232^§^
Metabolic defect	1 (0.0)	0 (0.0)	1.000^§^
Ear & facial defect	92 (0.1)	6 (0.2)	0.067^§^
Cleft lip and/or cleft palate	106 (0.1)	5 (0.1)	0.262^§^
Skin defect	7 (0.0)	0 (0.0)	1.000^§^
Others	37 (0.0)	2 (0.1)	0.316^§^

*The total numbers of pregnancies in the spontaneous conception group for maternal age at delivery and location were 109,538 and 109509, respectively.

^#^The total number of births from pregnancy by spontaneous conception was 111,050, and the total number of births from pregnancy by ART-assisted conception was 3,336.

^§^P values for Fisher’s exact tests.

^¶^P values for Student’s t/t’ tests.

Among the newborns included in the analysis, 1,108 infants (1.0%) were diagnosed with at least one birth defect. The infants conceived by ART had an increased likelihood of developing any birth defect compared to infants conceived by natural means (2.3%, vs. 0.9%, P < 0.001); the crude OR was 2.52 (95% CI, 2.00 to 3.18), and the risk was reduced but remained significant after adjusting for potential confounders, with an adjusted OR of 2.10 (1.63 to 2.69). The association between assisted conception and any birth defect among infants from singleton births was similar to that among all newborns, with crude and adjusted ORs of 2.38 (1.34 to 4.21) and 2.17 (1.51 to 3.13), respectively. However, the relationship between ART and any birth defect among infants from multiple births was weaker than that among infants from singleton births, with crude and adjusted ORs of 1.49 (1.01 to 2.22) and 1.44 (0.95 to 2.17), respectively (Table 2).

**Table 2:** Odds ratios for stillbirths and birth defects among infants from assisted conception according to multiplicity.

Outcomes	All births		Singleton births		Multiple births	
	Unadjusted odds ratio	Adjusted odds ratio^**#**^	Unadjusted odds ratio	Adjusted odds ratio^**#**^	Unadjusted odds ratio	Adjusted odds ratio^**#**^
Stillbirth	3.70 (2.59 to 5.28)	0.96 (0.64 to 1.44)	1.43 (0.59 to 3.47)	1.58 (0.62 to 4.02)	0.71 (0.38 to 1.32)	0.83 (0.53 to 1.32)
Birth defect^§^						
Any defect	2.52 (2.00 to 3.18)	2.10 (1.63 to 2.69)	2.38 (1.34 to 4.21)	2.17 (1.51 to 3.13)	1.49 (1.01 to 2.22)	1.44 (0.95 to 2.17)
Cardiovascular defect	3.66 (2.34 to 5.70)	2.13 (1.28 to 3.55)	1.89 (0.78 to 4.60)	1.93 (0.74 to 4.99)	1.68 (0.86 to 3.30)	1.36 (0.69 to 2.67)
Musculoskeletal defect	1.71 (1.09 to 2.68)	1.59 (0.95 to 2.67)	2.30 (1.32 to 4.01)	2.38 (1.33 to 4.24)	0.78 (0.32 to 1.87)	0.77 (0.31 to 1.91)
Urogenital defect	3.62 (2.19 to 5.97)	3.44 (1.99 to 5.96)	4.09 (2.08 to 8.04)	3.78 (1.87 to 7.64)	1.26 (0.51 to 3.08)	1.44 (0.57 to 3.65)
Gastrointestinal defect	3.62 (1.44 to 9.11)	3.39 (1.09 to 10.57)	2.92 (0.71 to 12.05)	3.33 (0.78 to 14.23)	5.67 (0.59 to 54.47)	4.94 (0.49 to 49.79)
Central nervous system defect	7.40 (1.60 to 34.23)	1.37 (0.24 to 7.90)	—	—	1.26 (0.21 to 7.52)	1.02 (0.16 to 6.50)
Respiratory defect	16.64 (3.05 to 90.88)	5.69 (0.61 to 53.28)	21.91 (2.28 to 210.71)	11.19 (1.48 to 84.56)	1.89 (0.12 to 30.16)	2.18 (0.14 to 34.97)
Chromosomal defect	1.85 (0.58 to 5.91)	2.16 (0.58 to 7.97)	1.22 (0.17 to 8.80)	1.05 (0.13 to 8.46)	—	—
Ear & facial defect	2.17 (0.95 to 4.96)	1.96 (0.75 to 5.11)	2.22 (0.70 to 7.01)	1.97 (0.62 to 6.31)	1.89 (0.38 to 9.35)	1.52 (0.30 to 7.77)
Cleft lip and/or cleft palate	1.57 (0.64 to 3.85)	1.10 (0.40 to 3.02)	0.64 (0.09 to 4.62)	0.57 (0.08 to 3.98)	1.89 (0.47 to 7.55)	2.57 (0.52 to 12.62)
Others	1.80 (0.43 to 7.46)	1.38 (0.28 to 6.78)	—	—	—	—

**The odds ratios reflect the risk of birth defects for the assisted conception group versus the spontaneous conception group and were calculated by logistic generalized estimating equations to control for the effect of the clustering of births within women.

^#^Maternal age at delivery (≤24, 25–34, and ≥35), residence (Shanghai, other provinces), gravidity (number), history of abnormal pregnancy (yes and no), parity (nulliparous and pluripara), birth weight (g) and year, maternal disease in pregnancy (pre-existing hypertension and diabetes, gestational hypertension, gestational diabetes mellitus, or anaemia), and foetal gender were further adjusted for in the generalized estimating equations.

^§^Odds ratios for metabolic defects and skin deformity were not shown due to the absence of defect cases in the spontaneous and assisted conception groups.

Infants conceived by ART also had a significantly increased likelihood of subcategories of cardiovascular, urogenital, and gastrointestinal defects compared to all infants (ORs ranging from 2.13 to 3.44). However, no significant associations were found between ART and musculoskeletal and respiratory defects, central nervous system defects, chromosomal defects, ear and facial deformations, and cleft lip and/or cleft palate. In the strata of multiplicity analyses, the associations between ART and cardiovascular and gastrointestinal defects disappeared, whereas ART was significantly associated with musculoskeletal and respiratory defects (ORs of 2.38 and 11.19, respectively) among singleton births, but no significant associations were observed between ART and all subcategories of birth defects in the subgroup of multiple births (Table 2).

### ART and stillbirth

The incidence of stillbirth among the women in the assisted conception group was significantly higher than that among the women in the spontaneous conception group (34 cases, 1.0% vs. 308 cases, 0.3%, P < 0.001). Compared with the infants conceived naturally, the ART-conceived infants had a higher likelihood of stillbirth, with a crude OR of 3.70 (95% CI, 2.59 to 5.28). However, no significant association between ART and the risk of stillbirth was observed after adjusting for potential confounders in all births (OR = 0.96, 95% CI, 0.64 to 1.44) or in the subgroups of singleton and multiple births (Table 2).

### Multiple gestations, ART, and birth defects

ART was associated with a significant increase in the likelihood of birth defects in the logistic regression analysis using path analysis models, with an adjusted OR (total effect of ART on birth defects) of 2.22 (95% CI, 1.75 to 2.81). The ART-conceived newborns would have had a 1.64-higher likelihood of birth defects than spontaneously conceived births if the risk of multiple pregnancies had been kept constant at the level of spontaneously conceived births (OR = 1.64, 95% CI, 1.27 to 2.12, direct effect of ART on birth defects). Approximately 37.75% of the total effect of ART on birth defects was mediated by the association between ART and multiple pregnancies (indirect effect), with an adjusted OR of 1.35 (94% CI, 1.19 to 1.53) (Table 3).

**Table 3:** Distribution of the total effect of ART on the likelihood of birth defects into direct and indirect effects mediated by multiple pregnancies.

Mode of conception		Total effect		Direct effect		Indirect effect		Estimated size of the indirect effect
		OR	95% CI	OR	95% CI	OR	95% CI	
Spontaneous conception		1.00		1.00		1.00		
Assisted conception	Unadjusted model	2.53	1.97 to 3.25	1.82	1.33 to 2.49	1.39	1.20 to 1.61	35.23%
	Adjusted model*	2.22	1.75 to 2.81	1.64	1.27 to 2.12	1.35	1.19 to 1.53	37.75%

*Adjustments were applied for maternal age at delivery (≤24, 25–34, and ≥35), residence (Shanghai, other provinces), gravidity (number), history of abnormal pregnancy (yes and no), parity (nulliparous and pluripara), birth weight (g) and year, maternal disease in pregnancy (pre-existing hypertension and diabetes, gestational hypertension, gestational diabetes mellitus, or anaemia) and foetal gender in the logistic regression analysis using path analysis models.

Multiple gestations were associated with a significantly increased likelihood of birth defects among infants conceived by spontaneous conception, with prevalence rates of birth defects among infants from singleton, twin, and triplet births of 0.9%, 1.7%, and 3.3%, respectively. The adjusted ORs of birth defects for twin and triplet infants were 1.86 (1.37 to 2.52) and 3.37 (1.13 to 10.12), respectively, compared with those for singleton infants (Table 4). However, the relationship between multiple gestations and birth defects was not statistically significant among the infants conceived by ART (Table 4). The results had statistical powers of 98.9% and 100% to detect effect sizes of 1.16 and 1.48 using sample sizes of 3,288 and 1,698 with a 5% two-sided significance level, respectively. Compared with spontaneous singleton infants, the combined effect of ART and twins on the risk of birth defects was lower than that of the sum of the individual effects of ART and twins in the interaction analysis, with an adjusted OR of 0.54 (0.32 to 0.92) (Table 5).

**Table 4:** Prevalence and odds ratios for birth defects across the groups of multiplicity according to the mode of conception.

Mode of conception	Multiplicity	No. of births	No. of defects	Prevalence (%)	P value	Odds ratio [95% CI]	
						Crude	Adjusted^**#**^
Assisted conception	Singleton	1,644	33	2.0	0.456	1.00	1.00
	Twin	1,644	42	2.6		1.28 [0.81 to 2.02]	1.16 [0.50 to 2.67]
	Triplets	54	2	3.7		1.88 [0.46 to 7.61]	1.48 [0.34 to 6.46]
Spontaneous conception	Singleton	107,957	975	0.9*	<0.001	1.00	1.00
	Twin	3,082	52	1.7		1.88 [1.43 to 2.49]	1.86 [1.37 to 2.52]
	Triplets	120	4	3.3		3.78 [1.44 to 9.92]	3.37 [1.13 to 10.12]

*P value < 0.05 for the chi-square test comparing the prevalence of birth defects among singleton births between the groups of assisted conception and spontaneous conception.

^#^Analyses were adjusted for maternal age at delivery (≤24, 25–34, and ≥35), residence (Shanghai, other provinces), gravidity (number), history of abnormal pregnancy (yes and no), parity (nulliparous and pluripara), birth weight (g) and year, maternal disease in pregnancy (pre-existing hypertension and diabetes, gestational hypertension, gestational diabetes mellitus, or anaemia) and foetal gender in generalized estimating equations models.

**Table 5:** Interaction between multiplicity and mode of conception in the risk of birth defects.

	Crude OR	95% CI		P value	Adjusted OR*	95% CI		P value
Multiplicity								
Singleton	1.00				1.00			
Twins	8.19	6.12	10.95	<0.001	8.00	5.78	11.07	<0.001
Triplets	31.49	11.72	84.64	<0.001	27.70	10.28	74.58	<0.001
Mode of conception								
Spontaneous conception	1.00				1.00			
Assisted conception	2.77	1.96	3.92	<0.001	2.33	1.63	3.32	<0.001
Interaction term (ART × multiplicity)								
Spontaneous conception × singleton	1.00				1.00			
ART × twins	0.53	0.31	0.90	0.020	0.54	0.32	0.92	0.024
ART × triplets	0.88	0.15	4.96	0.880	1.04	0.18	5.91	0.963

*Analyses were adjusted for maternal age at delivery (≤24, 25–34, and ≥35), residence (Shanghai, other provinces), gravidity (number), history of abnormal pregnancy (yes and no), parity (nulliparous and pluripara), birth weight (g) and year, maternal disease in pregnancy (pre-existing hypertension and diabetes, gestational hypertension, gestational diabetes mellitus, or anaemia) and foetal gender in Cox regression models.

## Discussion

In this large retrospective study of pregnant Chinese women between 2006 and 2016, we verified that infants conceived by ART have an increased risk of birth defects compared with those conceived spontaneously among all births and among singleton births. Multiple gestations have an increased risk of birth defects among infants from spontaneous conception but not among those from ART-assisted conception. Approximately 37.75% of the total effect of ART on birth defects may be mediated by multiple pregnancies. Furthermore, the combined effect of ART and twins on birth defects was lower than the sum of the individual effects of ART and twins.

The relationship between ART and birth defects has been controversial because several previous studies have found that the increased risk of birth defects after ART is attributed to maternal characteristics related to infertility^13–16,29^. Nevertheless, similar to the findings of other previous studies^2,3,11,30^, we found that ART results in an increased risk of birth defects after controlling for the impacts of abnormal pregnancy history and perinatal complications. The association between ART and birth defects seems more pronounced in our study than that in previous studies (adjusted ORs for any defect ranging from 2.10 to 3.44 in the present study versus 1.16 to 1.30 in a previous study)^3^, which may suggest that in addition to the risk of birth defects from the induction of ovulation with medications during ART treatment (e.g., clomiphene citrate)^11,31^, additional variables may enhance the risk of birth defects among infants conceived by ART. Moreover, both the increased possibility of pollution in the process of ART due to its late development in Shanghai (the first infant conceived by IVF was not delivered until 1995 in eastern China)^32^ and a higher baseline risk of birth defects among pregnancies conceived from ART due to differences in race^2,33^ may increase the risk of birth defects among infants from ART-assisted pregnancies in our study.

We also found associations between ART and cardiovascular, musculoskeletal, urogenital, gastrointestinal, and respiratory defects among all births and singleton births, which have been previously reported^11,27,34–36^. However, no significant associations between ART and other system defects were observed, which may be due to the low frequency of these outcomes in eastern China^37^. Moreover, the risk of ART and certain defects, such as central nervous defects, may be counteracted by the protective effect of free folic acid supplementation for cerebral palsy, which began in 2009 in China^38,39^, and by early intervention (e.g., induced abortions for anencephaly or severely multi-malformed infants) as a result of ultrasound detection^38^.

Several potential mechanisms may be involved in the causal pathway between ART and birth defects. Previous studies have reported that ART may cause uncontrolled propagation of aneuploid cells in human oocytes and embryos^2^, increase the risks of gene mutation in offspring^40^, and cause epigenetic errors, such as defects in DNA methylation and imprinting, thus resulting in an increased risk of birth defects^41–43^.

No significant association between ART and stillbirth was found after adjusting for potential confounders in all births. However, different impacts of ART on the risk of stillbirth were observed in the subgroups of singleton and multiple births. For example, ART was associated with an increased risk of stillbirth among singletons, whereas multiple births from ART were associated with a lower risk of stillbirth compared to those from spontaneous conception. Therefore, the results should be interpreted cautiously, and additional studies are required to determine the association of ART with stillbirth in China.

Infants from multiple gestations are at a higher risk of birth defects than infants from singleton gestations among infants from spontaneous conceptions but not among those from ART-assisted conceptions. The difference between infants from singleton and multiple births may reflect a greater increase in the risk of birth defects among naturally conceived infants from multiple gestations than that among infants from multiple gestations conceived by ART when compared to singleton infants^44^.

In the logistic regression analyses using path-analysis models, we found that approximately 37.75% of the effect of ART on birth defects may be attributed to the higher risk of multiple pregnancies associated with ART. We further found a subtraction effect of ART and twins on birth defects, indicating that the combined effect of ART and twins was lower than the sum of the individual effects of ART and twins. Twins conceived by ART are much more likely to be dizygotic than twins conceived spontaneously^11,45,46^ (94.1% of ART-conceived twins vs. 69.6% of naturally conceived twins in the present study, P < 0.001). Given that dizygotic twins are at a lower risk for birth defects than monozygotic twins, this could explain why the likelihood of birth defects was not significantly different between twins conceived by ART and twins conceived spontaneously.

These findings have important consequences for public health. Women may choose to transfer two embryos since ART-conceived twins have a lower combined risk for birth defects than the sum of the individual effects of ART and twins. Furthermore, women may not worry about the additional risk of stillbirth associated with ART but may instead focus more on organs at a high risk of birth defects, including cardiovascular, musculoskeletal, urogenital, gastrointestinal, and respiratory organs. ART staff and obstetricians should also direct more attention towards the screening of organs at a high risk in foetuses to detect birth defects earlier and implement appropriate intervention measures.

Nevertheless, our study has several limitations. First, this study lacks information regarding subfertility and infertility treatments for infertile women during pregnancy. Consequently, we were not able to rule out impact of infertility on the association between ART and birth defects. Second, the low prevalence of birth defects in our study, which may be attributed to the fact that we did not include pregnant women who had terminated a pregnancy because of foetal birth defects detected during pregnancy, may limit the interpretation of our results. For example, pregnancies conceived by ART may be monitored frequently and therefore reflect higher detection rates for birth defects compared to pregnancies from natural conceptions, resulting in overestimation of the relationship between ART and birth defects. Furthermore, potential missed birth defect diagnoses after birth may partly explain the low prevalence of birth defects in our study since we only included infants who had been diagnosed within 7 days of birth and had been confirmed within 42 days among those with suspected birth defects after hospital discharge.

Third, limited by the study design, we were not able to include in the analyses all potential confounders associated with a higher risk of birth defects, such as environmental exposures and risk behaviours (alcohol and tobacco use)^47,48^, which may lead to an information bias and reduce the internal validity of the study results. Finally, the subjects were selected from a high-level specialist hospital, which may limit the generalizability of the sample (subjects may have higher education and/or income levels) and the generalizability of the results.

In this large retrospective study of Chinese pregnant women and newborns, we found that ART was associated with an increased risk of birth defects, and approximately 37.75% of the total effect of ART on birth defects may be mediated by the higher risk of multiple pregnancies associated with ART. We first found that ART-conceived twins had a lower risk of birth defects than the sum of the individual effects of ART and twins. The results clearly show the association between ART and the risk of birth defects, and part of the effect of ART on birth defects may be due to multiple pregnancies. These findings may provide guidance to couples and obstetricians in selecting the number of pregnancies and in identifying organs at a high risk of birth defects.

## Methods

### Sources of data

A retrospective cohort study was conducted in Shanghai, China, in 2017. Pregnant women and their infants born at the Obstetrics and Gynecology Hospital of Fudan University between 2006 and 2016 were recruited for the study. Whether the pregnancies were conceived naturally or by ART was self-reported by the pregnant women. Pregnancies conceived by ART were defined as pregnancies established through ART, including IVF and ICSI. The applicability and technical level of ART did not change between 2006 and 2016.

All newborns were followed up for 7 days after birth. Newborns suspected of having birth defects but without a diagnosis at 7 days after birth were further followed up for 42 days after birth through the Shanghai Perinatal Statistics Collection System (SPSCS), which requires notification of all live births and stillbirths from at least 28 weeks of gestation to 42 days after birth. Once birth defects were detected, a standard survey was conducted by a neonatal physician who was responsible for reporting the birth defect through the Chinese Birth Defect Surveillance Network. The data of the pregnant women and infants were extracted from the electronic medical record systems and were matched by their unique record numbers.

Basic information of the pregnant women, such as maternal age, gravidity, parity, abnormal pregnancy history and residence, were surveyed at their first visits. Maternal diseases in pregnancy, such as gestational diabetes, pre-existing diabetes, anaemia, hypertension, and pregnancy-induced hypertension, were diagnosed according to the corresponding criteria of the diseases based on measurements during the follow-up^16,29,49^. Neonatal birth outcomes, including birth weight, gender and birth year, were also collected before discharge from the hospital.

Birth defects were defined as functional and/or structural congenital abnormalities and were diagnosed by sonography, tandem mass spectrometry, X-rays, genetic tests or pathology within 7 days after birth. All congenital abnormalities are coded according to the International Classification of Diseases 10th Revision^2^. The diagnoses of congenital abnormalities included cardiovascular abnormalities (Q20-Q28), musculoskeletal abnormalities (Q65-Q79), urogenital abnormalities (Q50-56 and Q60-64), gastrointestinal abnormalities (Q38-Q45), central nervous system defect (Q00, Q05, and Q01/Q04/Q06/Q07), respiratory abnormalities (Q30-Q34), chromosomal abnormalities (Q90-Q99), metabolic defects (E71-75), ear and facial defects (Q11/Q12 and Q16-Q18), cleft lip and/or cleft palate (Q35-Q37), skin defects (Q80-Q84), and others (Q85-Q89). Potential confounding factors in the study included maternal age (≤24, 25–34, and ≥35 years), residence (shanghai or other provinces), gravidity (number), history of abnormal pregnancy (yes or no), parity (nulliparous or pluripara), maternal conditions during pregnancy (pre-existing hypertension and diabetes, gestational hypertension, gestational diabetes mellitus, and anaemia) and foetal gender, birth weight and year.

Approximately 10,000 pregnant women (ranging from 8,000 to 15,000) give birth at the Obstetrics and Gynecology Hospital of Fudan University every year, and we ultimately included more than 120,000 subjects in the study. Efforts were made to address potential biases of the study. For example, all pregnant women and newborns at the hospital between 2006 and 2016 were retrospectively investigated and included in the study, which may minimize the impact of loss to follow-up; new birth defect cases were confirmed by phone when the cases were reported in the SPSCS but not identified at the hospital; the birth years of newborns were included in the analyses to exclude the impact of potential incomparability of ART treatment; multivariate analysis methods were used to control for the impacts of potential confounding factors on the results.

The study was approved by the Ethics Committee of the Obstetrics and Gynecology Hospital of Fudan University (No. 2017-35) and was conducted in accordance with Chinese law and the Guidelines of the National Human Biomedical Research Policies. No informed consent was obtained from the patients because the study was retrospective.

### Statistical analysis

The proportions and means were used to describe the maternal variables and pregnancy outcomes between the groups of assisted and spontaneous conception. Chi-square tests or Fisher’s exact tests were used to compare proportions between the groups. Student’s t or t’ tests were conducted to compare the means between the groups. Crude odds ratios (ORs) and 95% confidence intervals (95% CIs) for the risks of birth defects and stillbirth among infants conceived by ART were estimated after adjusting for the correlations of multiple births among women using logistic generalized estimating equations (GEE)^3,16^. Adjusted ORs and the 95% CIs were evaluated after controlling for the impacts of the potential confounding factors mentioned above.

A large proportion of women who underwent ART-assisted conception had multiple gestations, which were thought to be a factor involved in the causal link between ART and birth defects^3,27^. A logistic regression analysis using a path analysis model was conducted to explain the extent to which the total effect of ART on birth defects may be mediated by the association between ART and multiple gestations (indirect effect)^50–52^. Stratification analyses were performed to compare the risk of birth defects across the groups of multiplicity according to strata of the mode of conception. Considering that the sample size of ART-conceived births used to investigate the relationship between multiple pregnancies and the risk of birth defects was small (3,342 ART-conceived-births in total), we calculated the statistical power to measure the effectiveness of the results. Interaction indicates that the effect of one risk factor on a certain disease outcome is different across the strata of another risk factor, or vice versa^53^. Therefore, interaction analysis was performed to estimate the combined effect of ART and twins or triplets compared with the sum of the individual effects of ART and twins or triplets using Cox regression models. An OR of the interaction term less than 1.00 indicates that the risk of the combined effect of ART and twins or triplets on birth defects was lower than that of the sum of the individual effects of ART and twins or triplets.

The missing rates for each variable are very low (ranging from 0% to 0.12%). Therefore, we excluded infants born to pregnant women who had any missing data for the variables in the analysis (0.14% of the births). Pathway analysis and statistical power calculations were performed with Stata 12 software (StataCorp, Texas, USA). All other statistical tests were conducted using IBM SPSS Statistics version 22.0 (IBM Corp., Armonk, NY, USA). A P value < 0.05 was considered statistically significant.
